# Time-Resolved Fluorescence Detection of Nicked DNA via Site-Specific Stacking of Sulfo-Cy3: The Role of Charge, Polarity, Linker, and Sequence Context

**DOI:** 10.1021/acs.jpcb.5c03845

**Published:** 2025-10-28

**Authors:** Raul Berrocal-Martin, Henry G. Sansom, Katalin Orosz, Max B. Paterson, Brian O. Smith, Steven W. Magennis

**Affiliations:** † School of Chemistry, 3526 University of Glasgow, Joseph Black Building, University Avenue, Glasgow G12 8QQ, U.K.; ‡ School of Molecular Biosciences, University of Glasgow, Joseph Black Building, University Avenue, Glasgow G12 8QQ, U.K.

## Abstract

Nicked DNA can result from damage or cellular processing and is also prevalent in DNA nanostructures, so sensitive methods to probe the generation and location of nicks are desirable. It was found recently that sulfo-Cy3 (sCy3), a disulfonated version of Cy3, could stack in a nick of double-stranded DNA (dsDNA) when attached to a thymidine on the 3′ end of one strand, resulting in an increase in fluorescence brightness. Here, we have performed a systematic time-resolved fluorescence study of the stacking of sCy3 on nicked DNA. We have varied the labeling polarity (5′ vs 3′), linker (dT vs phosphate), labeled nucleotide (A, C, T, or G), DNA structure (nicked dsDNA, dsDNA, single-stranded DNA, and dsDNA with a single-strand overhang), and NaCl concentration. We also studied the effect of switching the negatively charged sCy3 to the positively charged Cy3. We have shown that the site-specific stacking and fluorescence modulation of sCy3 on nicked DNA is a general phenomenon that could find application in time-resolved assays of DNA processes such as annealing, strand displacement, and enzymatic nicking.

## Graphical abstract

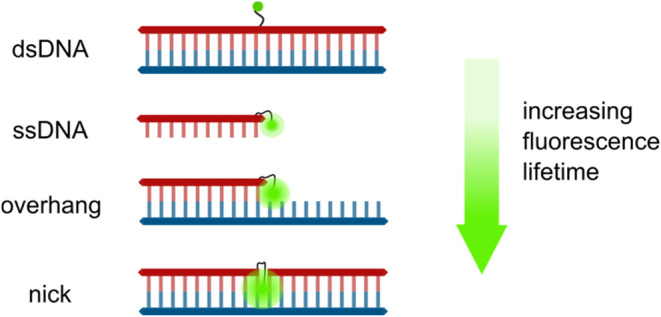

## Introduction

Nicks in DNA, also called single-strand breaks, are the most common form of DNA damage.^1^ They are also generated or serve as markers during many biological processes, including replication, recombination, and repair,^1,2^ while the topological freedom of nicked DNA is exploited by type I DNA topoisomerases to increase or decrease superhelical tension.^3^ Nicked DNA duplexes adopt a structure similar to intact DNA,^4,5^ with base-stacking interactions maintaining the overall structure while also providing more conformational freedom than intact DNA.^6^ Nicks are also central to the construction and application of DNA nanostructures.^7^ Therefore, sensitive methods to probe the location and generation of nicks are desirable. In this work, we explore an approach to nick detection using the stacking of a fluorescent cyanine label.

Carbocyanine fluorophores, such as Cy3 and Cy5, and their structural variants are important biological labels due to their brightness, photostability, and versatile chemical reactivity; applications include single-molecule detection, PCR, and super-resolution microscopy.^8–13^ Central to the photophysical and photochemical properties of these dyes is their ability to undergo excited-state photoisomerization from a highly fluorescent trans state to a weakly fluorescent cis state. This process, which is now well understood,^14–20^ is extremely sensitive to bulk viscosity and temperature,^21,22^ as well as interactions of the dye with its local environment, and has been exploited in a range of biophysical assays.^20^ A novel biological application of this environmental sensitivity resulted from the work of Kozlov and Lohman,^23^ who studied the interaction of the *E. coli* single-strand binding protein with a Cy3-labeled single-stranded DNA (ssDNA) via changes in Cy3 fluorescence. This was followed by the work of Xie^24^ and Ha laboratories,^25^ who introduced single-molecule assays in which fluorescence intensities of a Cy3-labeled DNA were modulated by the binding of DNA-binding proteins in the vicinity of the fluorophore; this was termed protein-induced fluorescence enhancement (PIFE) by Myong et al.^25^ As discussed in detail recently, this modulation is not restricted to interactions with proteins, and it has been proposed that the PIFE acronym be modified to photoisomerization-related fluorescence enhancement in order to reflect its wider applicability.^20^

In particular, it is well-known that cyanine dyes are strongly affected by their attachment to nucleic acids. Norman, Lilley, and co-workers showed that Cy3 and Cy5 attached to the 5′ end of one strand in a DNA duplex were able to stack on the end basepair;^26,27^ this was also observed for the more water-soluble sulfonated variants and for different linker lengths.^28,29^ This stacking leads to thermodynamic stabilization of the DNA,^30^ and has implications for the use of cyanine dyes as FRET probes due to reduced mobility.^27,31^ The photophysical properties of cyanine dyes interacting with DNA were first studied in detail by Levitus and co-workers, demonstrating that local interactions of the disulfonated version Cy3, referred to here as sulfo-Cy3 (sCy3), covalently attached to single-stranded DNA (ssDNA) and double-stranded DNA (dsDNA), can lead to large changes in fluorescence properties, strongly dependent on the structural context.^32^ The fluorescence modulation was shown to be due to microenvironmental control of photoisomerization.^32^

Interactions of free Cy3 or sCy3 with nucleoside monophosphates demonstrated a dependence of stacking on the base, with stronger interactions for purines than pyrimidines, attributed to π–π interactions.^33^ Stacking on ssDNA (for Cy3 on the 5′ end) also depends on sequence, with flexible sequences showing stronger interactions than rigid sequences (e.g., purine-rich tracts such as polyA) and with an influence of bases downstream of the attachment base.^34^ Computational studies of Cy3 attached to the 5′ end of dsDNA showed that the end-stacking is a dynamic process, dependent on the nucleotide to which the dye is attached.^35^ Comprehensive studies of the sequence dependence on Cy3 fluorescence intensity have been employed for ssDNA and dsDNA using microarrays with permutations of the bases adjacent to the end-labeled dye;^36–38^ these studies confirmed that adjacent purines increased the intensity and also that bases at least five nucleotides away had an influence.

Stacking in DNA is dominated by hydrophobic interactions,^39^ and the negatively charged sCy3 is generally used to minimize interactions with the DNA backbone. However, we recently reported that sCy3 attached to the 3′ end of a DNA hairpin could stack in a nicked DNA site-specifically, causing consequent increases in fluorescence intensity and lifetime that were associated with hairpin closing.^40^ The hairpin dynamics could be followed at the single-molecule level, giving the same information that can be obtained by single-molecule Förster resonance energy transfer (smFRET), but requiring only one fluorescent label.^41^ We called this stacking-induced fluorescence increase (SIFI), which can be considered as a site-specific example of nucleic acid-induced fluorescence enhancement (NAIFE).^20,42^ Both SIFI and NAIFE are examples of the PIFE mechanism discussed above, whereby the photoisomerization of the cyanine dye is modulated, resulting in changes in fluorescence intensity and lifetime. Stacking in a nick was also shown to be very sensitive to long-range perturbations. For example, structural changes propagated through a duplex DNA backbone were revealed by moving the location of an abasic site up to 20 base pairs away from a nick in which sCy3 could stack.^40^

Here, we explore the factors that control the site-specific stacking of sCy3 in nicked DNA by varying the polarity (3′ or 5′), linker (dT or phosphate), and attachment base (Figure 1) for different DNA structural contexts (ssDNA, dsDNA, overhang, and nick) and ionic strengths (by changing NaCl concentration). We also compare sCy3 with Cy3 for a range of structures. While this stacking-induced enhancement at nicks in DNA is shown to be a general phenomenon, there are large differences in the size of the enhancement, which are rationalized in the context of the earlier reports on Cy3/sCy3 interactions with DNA. Our results can be used for the design of new time-resolved fluorescence assays involving nicked DNA.

**Figure 1:**
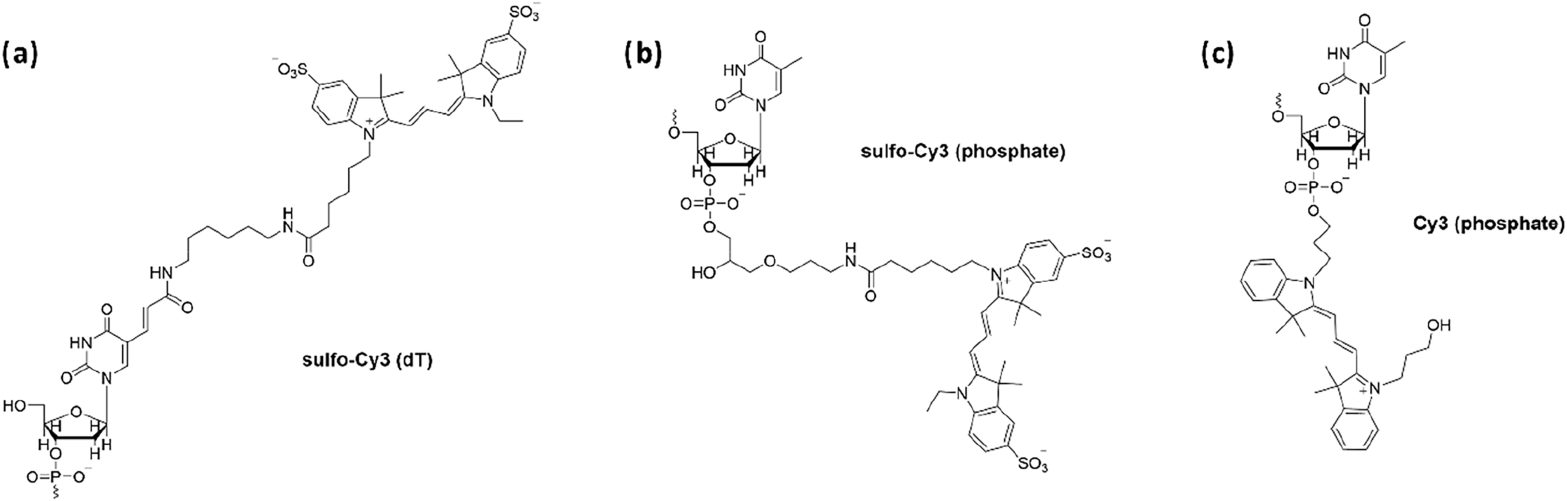
Structure of dyes and linkers used in this work. (a) sulfo-Cy3 (sCy3) attached via Ton the 3′ or 5′ end (5′ shown here). (b) sCy3 attached via phosphate with T at the 3′ end (also used with A, C, and G). (c) Cy3 attached via phosphate with T at the 3′ or 5′ end (3′ shown here).

## Materials and Methods

### Materials

NaCl (analytical reagent grade) was purchased from Fisher Scientific (Loughborough, UK). Tris base (BioUltra for luminescence, ≥99.8%) and Tris hydrochloride (reagent grade, ≥99%) were purchased from Sigma Life Sciences (Dorset, UK). All chemicals were used as received from the manufacturers.

The DNA oligonucleotides were synthesized and purified by IBA GmbH (Gottingen, Germany; double HPLC and PAGE, supplied lyophilized; the sequences are detailed in the Supporting Information).

The samples were dissolved in a buffer containing 20 mM Tris (pH 7.5) to obtain 100 μM DNA stock solutions. For DNA annealing, complementary strands (100 μM) were annealed in buffer (20 mM Tris-HCl/NaOH, 50 mM NaCl, pH 7.5) by heating them to 90 °C and then slowly cooled overnight. The ratio of labeled strand to the complementary strand was 1:2, ensuring that there was no free labeled strand in solution.

Fluorescence measurements were carried out in a 20 mM Tris buffer solution at pH 7.6 or 7.8, as indicated, at various NaCl concentrations and a temperature of 21 ± 1 °C.

### Absorption Spectroscopy

Absorption spectra were acquired on a Cary 60 spectrometer (Agilent Technologies, U.S.A.) at wavelengths from 200 to 800 nm. For quantum yield measurements, the absorbance was below 0.05 at the excitation wavelength.

### Steady-State and Time-Resolved Fluorescence Spectroscopy

Steady-state and time-resolved fluorescence were measured with a FluoTime 300 spectrometer (Picoquant, Germany) equipped with a hybrid PMT detector (PMA Hybrid 40, Picoquant). Fluorescence spectra were measured under magic angle conditions.

Fluorescence decays were recorded using time-correlated single-photon counting (TCSPC) with excitation from a pulsed supercontinuum fiber laser (Pulsed Fianium SC-400-4-PP, UK) coupled to a tunable Fianium SuperChrome filter (Fianium, UK) via optical fiber. The output of the SuperChrome system was coupled to the laser entry port of the FluoTime 300 spectrometer by optical fiber. Samples were excited with a repetition rate of 10 MHz (excitation wavelength: 532 nm with 10 nm bandpass). The fluorescence decay was collected over 104 ns, 13000 channels (8 ps/channel), and collected to a total of 10,000 counts in the peak channel (emission wavelength: 575 nm with 2 nm bandpass). Magic angle conditions were used throughout. The data were analyzed using FluoFit software (Picoquant).

Fluorescence decay curves were fitted by iterative reconvolution of the instrument response function (IRF) and the observed fluorescence decay, assuming a multiexponential decay function.

$$I\left(t\right)=\sum_{i=1}^{n}A_{i}\;\exp\left(\frac{-t}{\tau_{i}}\right)$$

where *I*(*t*) is the fluorescence intensity as a function of time, *t* (normalized to the intensity at *t* = 0); τ*_i_* is the fluorescence lifetime of the *i*th decay component; and *A_i_* is the fractional amplitude (A-factor) of that component. The quality of the fit was judged on the basis of the reduced chi-squared statistic, χ^2^, and the randomness of residuals.

Fluorescence quantum yields were measured for DNA in 20 mM Tris Buffer (pH 7.8; 0 mM NaCl) by the relative method using rhodamine 6G in ethanol (ϕ = 0.95) as a standard.^43^

### Accessible Volume Calculations

Accessible volume calculations were performed using the FRET positioning and screening (FPS) software described by Kalinin et al.^44^ The duplex structures were modeled as B-form DNA helices, using the x3dna software.^45^ The builder tool in Pymol^46^ was used to remove the phosphate group and create a nick. 3′ and 5′ ends were capped with hydroxyl groups to mimic the synthetic DNA samples used for experiments. The parameters for the dT-sulfo-Cy3 dye and linker used for the fluorescence experiments were taken directly from the procedure written by Kalinin et al.; C5 of the pyrimidine ring was used as the connecting atom of the labeled dT in the duplex structures. The resulting structures were visualized using Pymol. The two AV models were superimposed using the pair fit tool in Pymol.

## Results and Discussion

### Hairpins for Structural Characterization

In this section, we study a DNA hairpin as a control sample to benchmark the photophysical changes involved in stacking. We also establish the stability of the hairpin’s stem sequence, which forms the basis for the structures studied in the next section.

The stacking-induced fluorescence enhancement of sCy3, attached on the 3′ end via thymidine (named the dT linker here; Figure 1a), first reported by Morten et al., involved a DNA hairpin that closed to form a nicked, gapped, or overhang structure.^40^ Upon hairpin closing, the dye stacked and the fluorescence increased. Replacing sCy3 with Cy3B, which cannot undergo photoisomerization, resulted in no change in fluorescence intensity or lifetime upon hairpin closing.^40^ The same behavior was found when the DNA was immobilized on a surface for single-molecule fluorescence detection, indicating that the changes were intramolecular in origin and not due to the formation of aggregates.^40^ The hairpin structures in the original study contained six bp in the stem and underwent salt-dependent opening and closing. In this work, we first designed new DNA hairpins that have an additional two bases in the stem, increasing the thermodynamic stability of the closed hairpin (Figure 2a). In addition, the polyA hairpin loop is replaced by two hexaethylene glycol (HEG) linkers to reduce the overall size of the molecule and to form the desired nicked structure from a single DNA strand. The dye was placed next to the nick in either the 3′ or 5′ position. The sCy3 was attached to the 3′ end via a phosphate linker (Figure 1b) or the dT linker (Figure 1a), and to the 5′ end by dT. In a double-stranded DNA (dsDNA) control (Figure 2b), the dye was placed in the middle of duplex DNA on a dT, far from the blunt ends. We also studied the nicked hairpin that was reported previously (named H1 here) for comparison (Figure 2c).^40^

**Figure 2:**
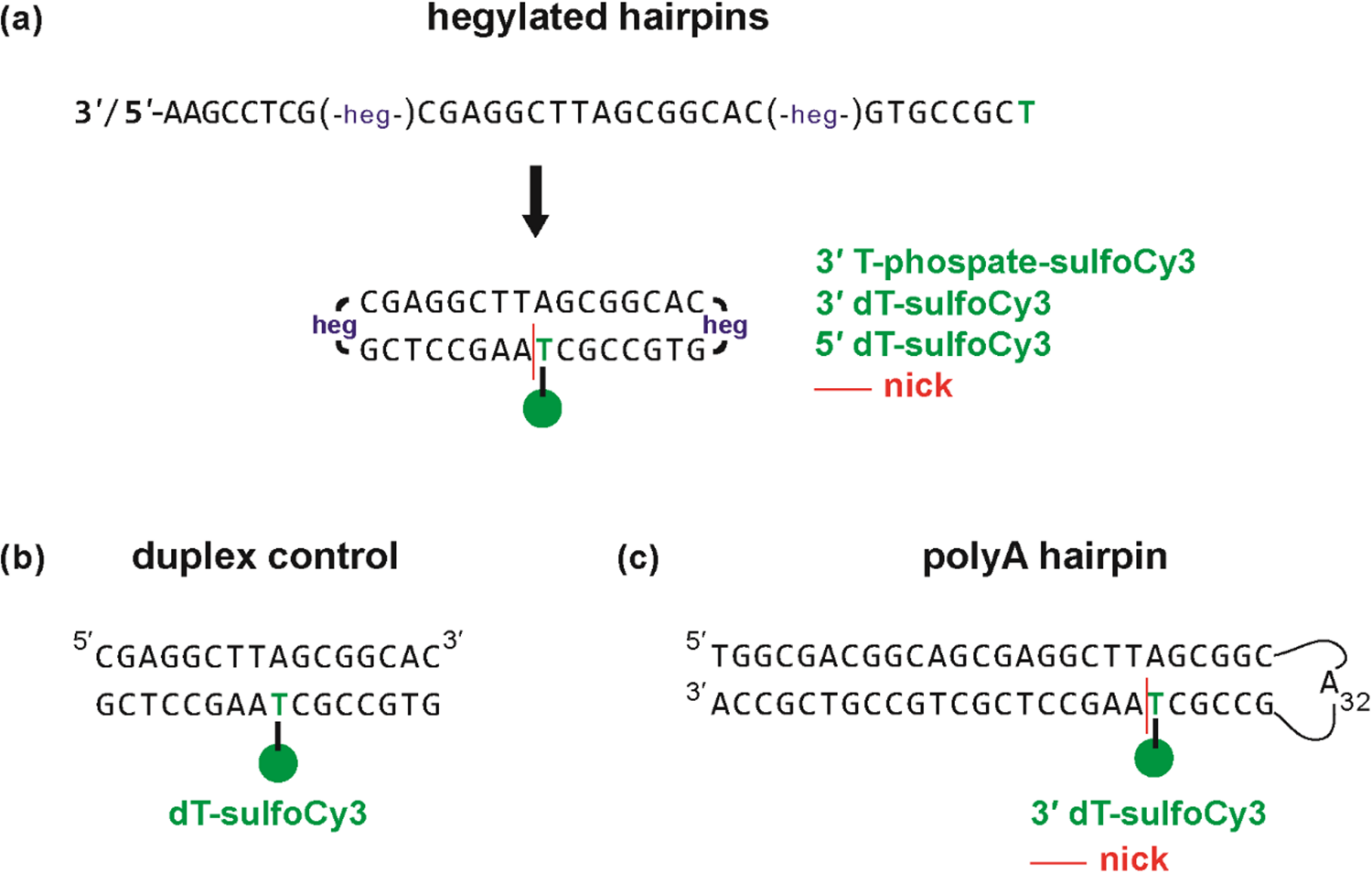
Hairpin and duplex DNA structures used as controls of sCy3 stacking. The cartoons represent the structure of (a) the HEG hairpins, (b) a duplex control, and (c) a polyA hairpin (H1) that was reported previously.^40^ The 3′ and 5′ hairpins have the same sequence but with reverse polarity. The 3′ hairpins have a sCy3 attached via either a phosphate or dT; the other samples shown here all have a sCy3 attached via dT.

We used TCSPC to measure the fluorescence lifetimes of the hairpins and the dsDNA control in a Tris buffer. As found previously,^32,40^ all fluorescence decays were multiexponential and were satisfactorily fit to three decay components. A long component of 1.5–2.5 ns lifetime is assigned to a highly stacked conformation, the short component of around 200 ps to free sCy3, and an intermediate of 0.6–1.1 ns is assigned to a somewhat conformationally restricted state, such as binding in the minor groove.^47^ The long component has a lifetime similar to that of Cy3B, which cannot photoisomerize.^32^ These states are dynamically interconverting, as shown by single-molecule experiments in which the signal is a weighted average of the three states.^40^

Representative fitted data at 20 mM NaCl highlight the key features of the data (Table 1). The 3′ HEG hairpins, with the sCy3 linked via phosphate or dT display, have the longest average lifetimes (1.48 and 1.36 ns, respectively) and similar weights of each decay component, with at least 40% of the dye in the stacked conformation and around 30% in the free conformation. In contrast, the 5′ sCy3 on dT has a much shorter average lifetime (0.55 ns), with a stacked population of only 9%, with 68% of the dye unstacked. Thus, the major contribution to the change in average lifetime is the change in population between fully stacked and unstacked conformations. The average lifetime of the dsDNA with internal dT labeling is only slightly shorter than the 5′-labeled hairpin at 0.33 ns. At 20 mM NaCl, the population of unstacked dye in the dsDNA is 82%. Despite the dye being internally labeled, located eight nucleotides from the duplex end, it still shows 3% of a long lifetime component, possibly due to intercalation or an intermolecular interaction.

**Table 1:** Representative Time-Resolved Fluorescence Decays of Hairpins and dsDNA Labeled with sCy3 (20 mM NaCl, 20 mM Tris, pH 7.6)

sample	τ_1_ (ns)	A_1_ (%)	τ_2_ (ns)	A_2_ (%)	τ_3_ (ns)	A_3_ (%)	τ_av_ (ns)
3′ HEG (phosphate) hairpin	2.29	47.6	1.12	29.1	0.29	23.3	1.48
3′ HEG (dT) hairpin	2.45	39.6	1.19	23.6	0.28	36.8	1.36
5′ HEG (dT) hairpin	2.04	9.4	0.85	22.4	0.25	68.2	0.55
dsDNA(dT)	1.65	2.65	0.62	15.2	0.23	82.2	0.33

We showed previously that the observed single-molecule brightness of sCy3 on a DNA hairpin was proportional to the average fluorescence lifetime of the bulk samples.^40^ To examine this relationship in more detail, we measured the fluorescence quantum yield for two of the HEG hairpins and the dsDNA control (Table 2). It is clear that the increase in lifetime for each sample is mirrored by a corresponding increase in quantum yield. The values of ϕ/τ_av_ are similar to the reported radiative rate constant for free sCy3 in Tris buffer at 22 °C, which was calculated to be 5 × 10^8^ s^–1^ using the measured monoexponential fluorescence lifetime of 0.18 ns and a fluorescence quantum yield of 0.09.^32^ This relationship between lifetime and quantum yield has been observed previously for sCy3 conjugated to DNA,^32^ and indicates an absence of dark states, which would reduce the quantum yield but not the lifetime.

**Table 2:** Average Lifetime vs Quantum Yield in Hairpins and dsDNA Labeled with sulfo-Cy3 (20 mM Tris, pH 7.8, 0 mM NaCl)^a^

sample	τ_av_ (ns)	ϕ	ϕ/τ_av_ (s^–1^)
3′ HEG (phosphate) hairpin	1.31	0.38	2.9 × 10^8^
5′ HEG (dT) hairpin	0.52	0.17	3.3 × 10^8^
dsDNA (dT)	0.31	0.11	3.5 × 10^8^

a The sample standard deviation from 3 independent measurements for the quantum yield was ≤7%. See Supplementary Tables 2–4 for the lifetime data.

The fluorescence lifetime measurements were recorded at different NaCl concentrations from 0–500 mM (Figure 3 and Supplementary Tables 1–4). For the original H1 hairpin,^40^ there is an increase in fluorescence lifetime with increasing salt concentration, due to salt-induced closing of the hairpin, as shown previously (Figure 3 and Supplementary Table 1). The population of the long lifetime increases with salt concentration from 11 to 34% while that of the short component decreases from 55 to 25%. This is attributed to the closing of the hairpin, which allows the stacking of sCy3 in the resulting nicked duplex. The population with an intermediate lifetime is almost constant at 35–40% for all salt concentrations. In contrast, there were no significant changes in lifetime with NaCl concentrations for the HEG hairpins or the duplex between 0–500 mM (Figure 3, and Supplementary Tables 2–4). Notably, the average lifetime for H1 at a high NaCl concentration, when it is fully closed, is similar to that of the HEG-3′ hairpin at all NaCl concentrations. This confirms that the HEG hairpins are closed at all salt concentrations due to the two extra base pairs in the stem, and that the sCy3 has the same stacking interaction with the nick in the DNA backbone as it has in the original H1 hairpin.

**Figure 3:**
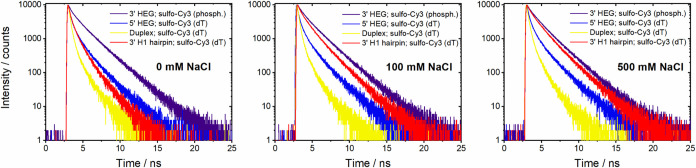
Time-resolved fluorescence decays of hairpins and duplex DNA at different NaCl concentrations. The buffer contains 20 mM Tris (pH 7.8) and either 0, 100, or 500 mM NaCl as indicated. See Supplementary Tables 1–4 for the fitted decay parameters.

It is clear that the average lifetime of the sCy3 in the 5′ position is always substantially lower than that for the 3′ HEG due to an increase in population of the unstacked state, primarily at the expense of the fully stacked conformation (Supplementary Tables 2 and 3). In an attempt to explain why sCy3 stacking is inhibited for 5′ in comparison to the 3′ labeling, we performed accessible volume (AV) calculations^44^ to map the possible dye locations when attached to DNA in the vicinity of a nick (Figure 4). We studied two duplexes that were identical to the 3′ and 5′ dT-labeled HEG hairpins discussed above, but without the HEG to join the strands together. Therefore, we required three strands to form these nicked duplexes (we discuss the fluorescence of these duplexes in the next section; see Figure 5 for the structures). AV calculations take into account the width and length of the linker and the radii of the dye. All possible locations of the dye constrained by the length of the linker are calculated, and those positions that result in a steric clash with the DNA are eliminated. The two AV-generated models for the 3′ and 5′ duplexes were superimposed for comparison (Figure 4), with the separate AV models shown in the Supporting Information (Figure S1). The AV for the 5′-labeled structure is ∼25% larger and extends further around the helix than for the 3′ structure. In spite of these differences, the AV does not explain why sCy3 is unable to access the nick. This suggests that the origin of the preferential stacking for 3′ is due to local steric constraints. Future experiments (e.g., NMR) or molecular modeling may provide insight into the nature of the stacking interactions. Nevertheless, the lack of a detailed atomistic model does not preclude its use in real applications. As discussed above, the dynamic nature of the Cy3-DNA interactions means that there is always a mixture of fully stacked, unstacked, and intermediate stacking, which is why we chose average fluorescence lifetime as a robust proxy for stacking.

**Figure 4:**
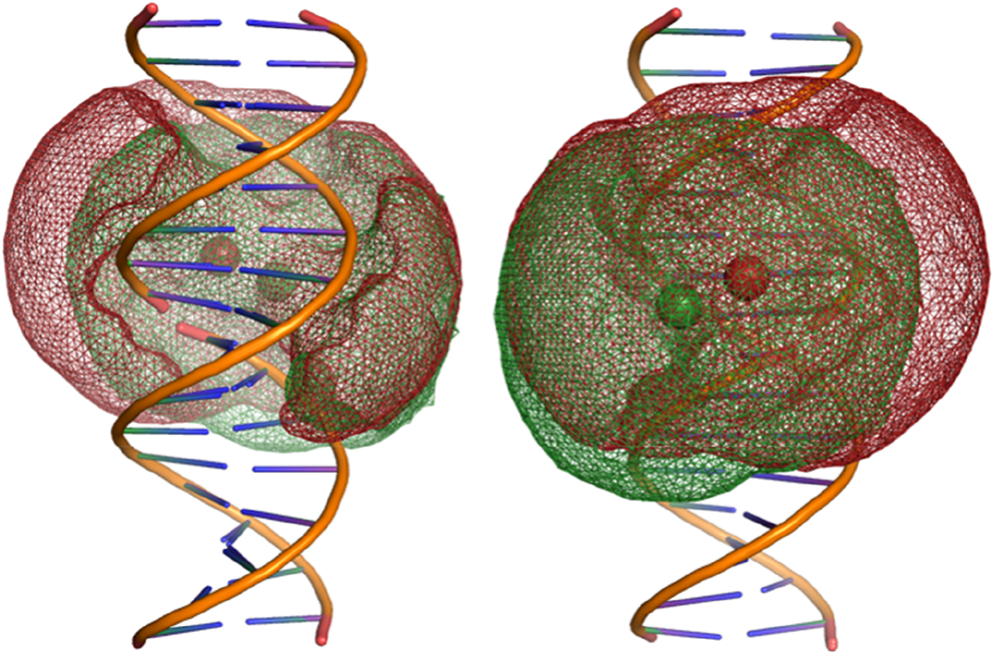
Accessible volume calculations for nicked duplexes. The structure with Cy3 attached via a 3′ dT at the nick is colored green; the structure with Cy3 attached to the 5′ dT at the nick is shown in red. The green and red spheres represent the mean dye positions for the 3′ and 5′ models, respectively. The image on the left shows the dye volume behind the nick site (as drawn), while the image on the right shows the dye volume in front of the nick.

**Figure 5:**
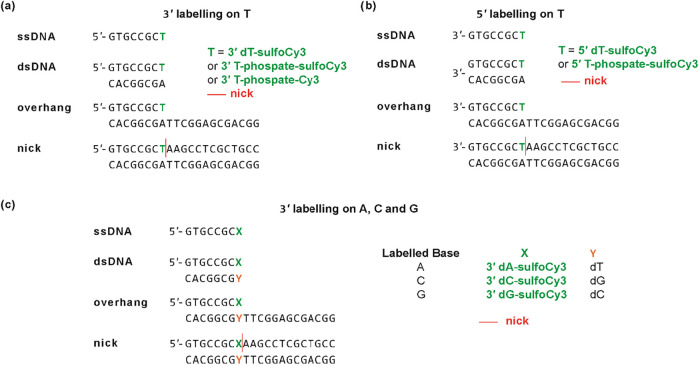
(a–c) DNA structures were designed to probe the interactions between sCy3/Cy3 and DNA.

### Design Parameters: Varying Polarity, Linker, Dye Charge, Identity of Labeled Nucleotide, and Salt Concentration

The experiments with the HEGylated hairpins (above) show that the extent of stacking in a nick depends upon whether the dye is labeled on 5′ or 3′. In order to predict or make use of stacking-induced fluorescence, it is important to understand all of the factors that contribute to this interaction. Therefore, we studied a series of samples (Figure 5) that were designed to investigate the effects of polarity (5′ vs 3′), linker (dT vs phosphate), charge on the dye (negative vs positive), and the nucleotide that the dye is attached to (A, T, C, or G). Each end-labeled oligo was studied as the ssDNA; as a blunt-ended dsDNA; as dsDNA with an overhang in the unlabeled strand where the dye is positioned at the ssDNA-dsDNA junction; and as nicked DNA with the dye adjacent to the nick, which is the same sequence context as for the HEGylated hairpins. These four sample types are referred to below as *ssDNA*, *dsDNA*, *overhang*, and *nick*, respectively. In total, 32 different DNA samples were studied. The sequences of all oligos and details of how they are combined to form each sample are given in the Supporting Information Table 5. In all structures, the dye is attached to an 8-mer oligo with the same sequence as the labeled end of the 3′ or 5′ HEG hairpins discussed in the preceding section. As shown above, these 8 bp sequences are stably annealed to their complement in the HEG hairpins at room temperature, ensuring that the double-stranded samples here (Figure 5) will also be stable.

#### 3′ Labeling on T

We investigated 3′ labeling to thymidine using the negatively charged sCy3 attached via the dT linker (Figure 1a), or the 3′ phosphate (Figure 1b). We also labeled the 3′ end with positively charged Cy3 to thymidine via the phosphate (Figure 1c). Time-resolved fluorescence of the ssDNA, dsDNA, overhang, and nick structures with each of these three labels was measured as a function of NaCl concentration (Figure 6). See Supplementary Tables 6–8 for representative data at each NaCl concentration (20, 50, and 200 mM)

**Figure 6:**
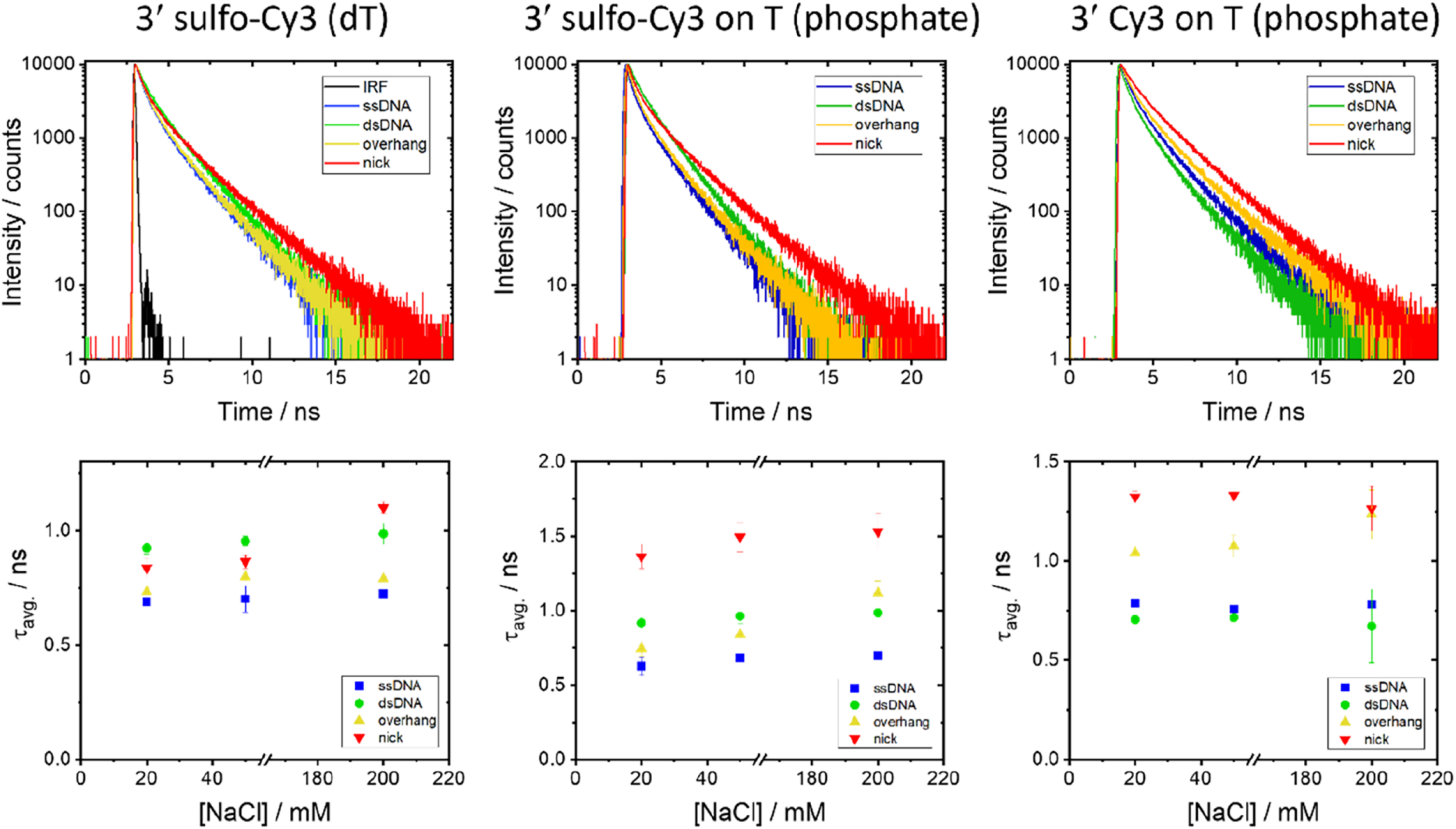
Time-resolved fluorescence decays of 3′-labeled samples at different NaCl concentrations. Decay curves (top) and average lifetimes (bottom) are shown for ssDNA, dsDNA, overhang, and nick samples labeled with sCy3 on T via base attachment (left), sCy3 on T via a phosphate (middle), and Cy3 on T via a phosphate (right). The buffer contains 20 mM Tris (pH 7.6) with 20, 50, or 200 mM NaCl; the decay curves shown are for 20 mM NaCl. The color coding is the same for the decays and the average lifetime graphs. Error bars for the average lifetime represent the sample standard deviation from the duplicate measurements. Note the different scales on the *y*-axes for the average lifetime plots. See Supplementary Tables 6–8 for representative decay parameters at each salt concentration. The intensity versus time graph for sCy3 on T via base attachment (top left panel) also includes the instrument response function (IRF), which was representative of all IRFs recorded in this work.

For all of the structures with 3′ labeling on T, three decay components were required for adequate fitting. The individual decay components have a similar range of values to the HEG hairpins, with the longest (0.63–1.53 ns) for the sCy3 attached via phosphate (Supplementary Table 7). Confirmation that the duplexes (Figure 5a) are structurally similar to the HEG hairpins (Figure 2a) is shown by comparing the average lifetimes for sCy3 attached via the phosphate (1.37 and 1.36 ns for the hairpin and duplex, respectively). There is a weak dependence on NaCl concentration for the negatively charged sCy3, presumably due to charge neutralization between the dye and DNA backbone; this is negligible for the positively charged Cy3. For all three dye-linker combinations, the average lifetime increases from ssDNA to overhang to nick, with the longest average lifetime of all samples for sCy3 linked via the phosphate (1.53 ns with 200 mM NaCl). In contrast, the average lifetimes when end-labeled on fully paired dsDNA have a strong dependence on the dye and linker. The positively charged Cy3 linked via phosphate has an average lifetime in the duplex that is shorter than ssDNA for all NaCl concentrations, whereas the sCy3 via phosphate has an average lifetime that is always higher than ssDNA at all concentrations and higher than that of the overhang for all but the highest NaCl concentration. For sCy3 attached via the thymine of dT, the average lifetime of the dsDNA sample is higher than that of the ssDNA, overhang, and nick at 20 and 50 mM NaCl, and is only slightly lower than the nick at 200 mM NaCl. We note that the linker used for attachment via the phosphate is shorter than when attaching via the base (Figure 1). Although the overall pattern of stacking is similar for the three linkers, some of the differences observed may be partly due to linker length, in addition to charge and labeling position (dT vs phosphate).

#### 5′ Labeling on T

For 5′ labeling, we studied the negatively charged sCy3 attached to T via the base (Figure 1a) or the positively charged Cy3 attached to T via the phosphate (Figure 1c). As for the 3′ samples above, we measured the fluorescence lifetimes for ssDNA, dsDNA, overhang, and nick structures with both of these labels as a function of NaCl concentration (Figure 7). See Supplementary Tables 9 and 10 for representative data at each NaCl concentration (20, 50, and 200 mM).

**Figure 7:**
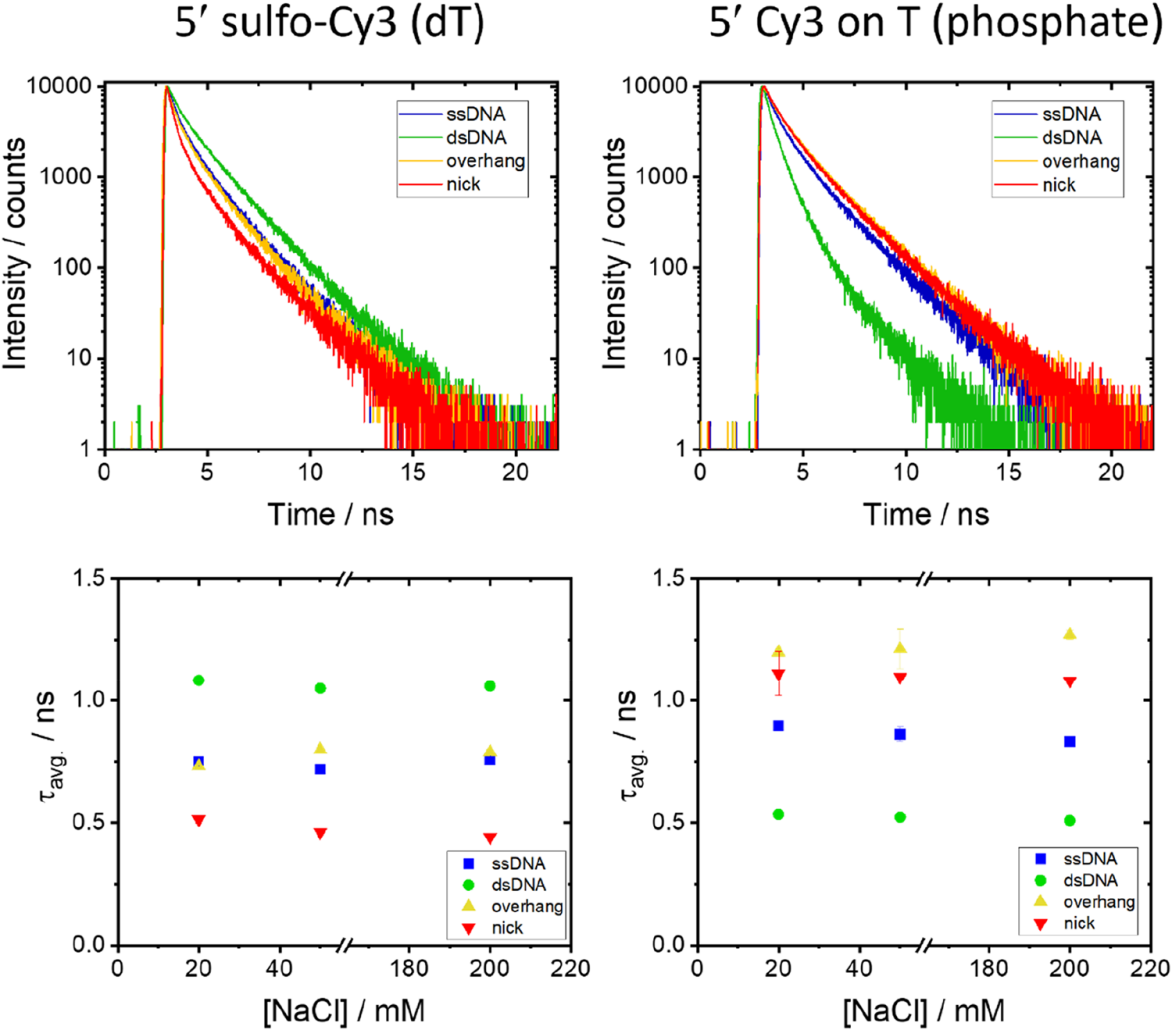
Time-resolved fluorescence decays of 5′-labeled samples at different NaCl concentrations. Decay curves (top) and average lifetimes (bottom) are shown for ssDNA, dsDNA, overhang, and nick samples labeled with sCy3 on T via base attachment (left) and Cy3 on T via a phosphate (right). The buffer contains 20 mM Tris (pH 7.6) with 20, 50, or 200 mM NaCl; the decay curves shown are for 20 mM NaCl. The color coding is the same for the decays and average lifetime graphs. Error bars for the average lifetime represent the sample standard deviation from duplicate measurements. See Supplementary Tables 9 and 10 for representative decay parameters at each salt concentration. See Figure 6 for a representative instrument response function (IRF).

The time-resolved fluorescence decays for the 5′ labels on T required fitting of three decay components that are in a similar range to the 3′ data. As with the 3′ samples, evidence that the duplexes (Figure 5b) are structurally similar to the HEG hairpins (Figure 2a) is shown by comparing the average lifetimes for sCy3 attached via the dT (0.50 and 0.51 ns for the hairpin and duplex, respectively). The average lifetime is almost independent of NaCl concentration. Like the 3′ sCy3 samples, the degree of stacking increases for the 5′ sCy3 (on dT) in the order of dsDNA > ssDNA ∼ overhang. In contrast to the 3′ data, the average lifetime for the nicked sample with sCy3 was the lowest of the four samples, whereas it was the second-highest or highest for both of the 3′ labels. In fact, the sample at 20 mM NaCl has the lowest average lifetime of all DNA samples measured at 0.44 ns. This dependence on labeling polarity matches that observed for the HEGylated hairpins. For the Cy3 label on the 5′ end, the trend in average lifetimes is closer to that observed for the 3′ sample, with the exception that stacking on the overhang is now greater than in the nick. We note that it was reported that when sCy3 is attached to ssDNA and dsDNA via a phosphate and short 3-carbon linker, the stacking was lower for the dsDNA;^32^ we see the same thing for Cy3 using the same linker. In contrast, we do not see this for the sCy3 attached via the T, highlighting the subtle effects of the dye-linker combination.^32^ Conversely, no difference in stacking was observed previously between the blunt-ended dsDNA and for an overhang with the Cy3 attached via phosphate,^32^ whereas we observe a clear difference.

#### 3′ Labeling on A, C, and G

As discussed, it is known that the stacking of Cy3 and sCy3 on ssDNA and dsDNA is strongly affected by the identity of the base to which they are attached. We investigated 3′ labeling using sCy3 attached to dA, dC, and dG via the phosphate (Figure 1a). We used the phosphate labeling here since it showed the biggest enhancement for nicked DNA when attached to T (Figure 6). As for the other samples, we measured the fluorescence lifetimes for ssDNA, dsDNA, overhang, and nick structures with each of these three labeled bases in a buffer containing 20 mM NaCl concentration (Figure 8). See Supplementary Table 11 for representative data.

**Figure 8:**
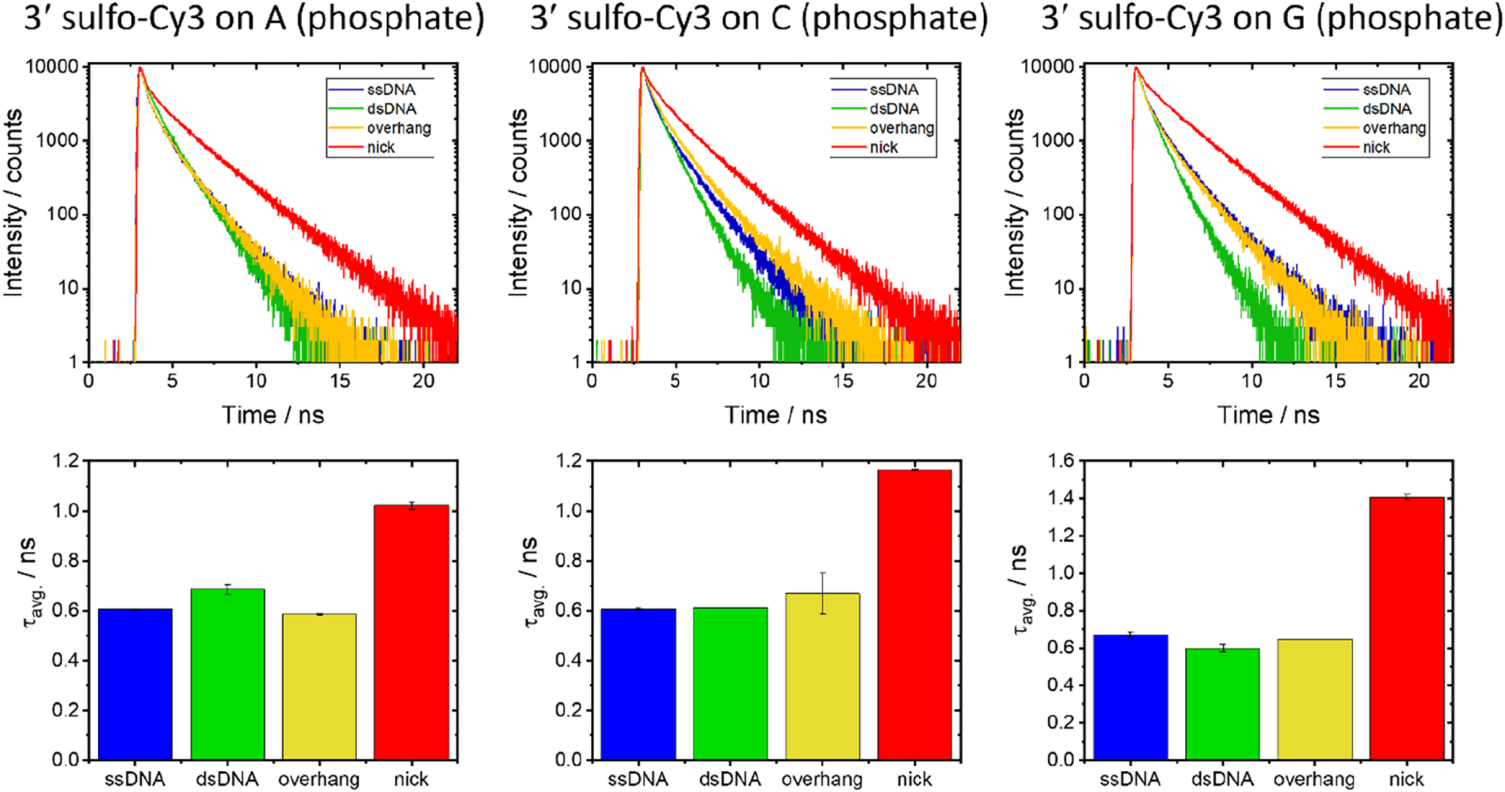
Time-resolved fluorescence decays of samples that are 3′-labeled via the phosphate with sulfo-Cy3 on dA, dC, and dG. Decay curves (top) and average lifetimes (bottom) are shown for ssDNA, dsDNA, overhang, and nick samples. The buffer contained 20 mM Tris (pH 7.6) with 20 mM NaCl. The color coding is the same for the decays and average lifetime graphs. Note the different scales on the *y*-axes for the average lifetime charts. Error bars for the average lifetime represent the sample standard deviation from duplicate measurements. See Supplementary Table 11 for representative decay parameters. See Figure 6 for a representative instrument response function (IRF).

The time-resolved decays are again fitted to three decay components. There is less variation in stacking for the different DNA structures than was observed at 20 mM NaCl for sCy3 labeled on the T via the phosphate (Figure 6). The average lifetimes for the ssDNA, dsDNA, and overhang structures are all at the lower end of the average lifetime range for all of the samples studied here, while the nicked samples show significantly greater stacking for all four bases.

The sequence dependence of stacking of Cy3 on the ends of ssDNA and dsDNA was attributed previously to the flexibility of the DNA and to the proximity of purines.^33–38^ The general trend for stacking of Cy3 on the neighboring base in ssDNA and dsDNA was dG > dA > dT > dC. For sCy3 labeled on the 3′ end via the phosphate in 20 mM NaCl (Supplementary Tables 7 and 11), we find that stacking on ssDNA and dsDNA is almost independent of the end base, with the exception of T, where there is greater stacking for dsDNA. There is also little dependence on the attachment base for the overhang structure. However, the nicked structures have particularly long average lifetimes for attachment to T and G (1.36 and 1.41 ns, respectively) with lower average lifetimes for A and C (1.02 and 1.17 ns, respectively).

## Conclusions

We performed a systematic study of the site-specific stacking of sCy3 and Cy3 in an adjacent nick. As discussed, stacking suppresses photoisomerization, resulting in enhanced fluorescence. By studying all samples using the same core DNA sequence under identical solution and measurement conditions, we were able to provide a detailed comparison of the key factors involved. We first studied a nicked hairpin that was formed from a single DNA strand with HEG spacers, where the nick was located in the middle of the 16 bp duplex. The use of a single strand ensured sample homogeneity. This hairpin was fully closed, even at low salt concentration, allowing us to maximize the stacking interaction. We used this duplex DNA sequence as the basis for all of the later sequences studied. By varying the choice of linker and/or dye, we have shown that it is possible to discriminate between any of the four DNA structures studied (ssDNA, dsDNA, overhang, and nicked). This provides an opportunity to monitor specific changes in the DNA structure. There are also dye-linker combinations where certain DNA structures have almost the same average lifetime, which could be used to ensure that the fluorescence brightness is independent of a particular structural change.

By using average fluorescence lifetime as a proxy for stacking efficiency, we have a robust method to evaluate stacking that is independent of factors such as sample concentration and illumination intensity. This would be an advantage for heterogeneous samples such as cells, and the bulk spectroscopy approach employed here could be easily extended to fluorescence lifetime imaging microscopy (FLIM). Over 3-fold increases in average lifetime have been recorded in the samples studied here, and we anticipate that this could be improved by examining the effect of DNA sequence beyond the attachment base. Future structural studies of the precise nature of the stacking interactions may also help to optimize the magnitude and selectivity of fluorescence enhancements. Many time-resolved fluorescence assays can be envisaged, both *in vitro* and *in vivo*, that would take advantage of the presence or absence of nicked DNA. These include the real-time monitoring of annealing, strand displacement, and enzymatic nicking.

## Supplementary Material



## Data Availability

The data supporting this paper are contained in the Supporting Information.

## Notes

Sequences of DNA oligonucleotides and structures, Supplementary Tables 1–11, and Supplementary Figure 1 (PDF)
