# Supplementary material for: Time-Resolved Fluorescence Detection of Nicked DNA via Site-Specific Stacking of Sulfo-Cy3: The Role of Charge, Polarity, Linker, and Sequence Context
Source: J Phys Chem B. 2025 Oct 28;129(45):11626–35. doi: 10.1021/acs.jpcb.5c03845 (PMC12621249; doi:10.1021/acs.jpcb.5c03845)
Supplement: Supplementary file 1 [file jp5c03845_si_001.pdf]

Time-Resolved Fluorescence Detection of Nicked DNA via Site-Specific Stacking of Sulfo-Cy3: The Role of Charge, Polarity, Linker and Sequence Context

Raul Berrocal-Martin,[1] Henry G. Sansom,[1] Katalin Orosz,[1] Max B. Paterson,[1] Brian O. Smith[2] and Steven W. Magennis[1,*]

[1] School of Chemistry, University of Glasgow, Joseph Black Building, University Avenue, Glasgow G12 8QQ, UK

[2] School of Molecular Biosciences, University of Glasgow, Joseph Black Building, University Avenue, Glasgow, G12 8QQ, UK

* corresponding author: steven.magennis@glasgow.ac.uk

Supporting Information

This file contains:

1. Sequences of DNA oligonucleotides and structures

2. Tables S1-11

3. Figure S1

Sequences of DNA oligonucleotides and structures

1. DNA Oligonucleotides

DNA oligonucleotides with Cy3 fluorophores (sulfo-Cy3 via dT, sulfo-Cy3 via phosphate and neutral Cy3 via phosphate) were synthesized and labelled by IBA (Germany).

Hairpins for structural characterisation

HEG-5' Ph

5'- / AAG CCT CG(-heg-)C GAG GCT TAG CGG CAC(-heg-) GTG CCG C(T-phosphate-sulfoCy3) /-3'

HEG-3' dT

5'- / AAG CCT CG(-heg-)C GAG GCT TAG CGG CAC(-heg-) GTG CCG C(T-sulfoCy3) /-3'

HEG-5' dT

5'- / (T-sulfoCy3)CG CCG TG(-heg-)C ACG GCG ATT CGG AGC(-heg-) GCT CCG AA /-3'

Duplex

5'- / CGA GGC TTA GCG GCA C/-3'

5'- / GTG CCG C(T-sulfoCy3)A AGC CTC G/-3'

H1

5'- / biotin- TGG CGA CGG CAG CGA GGC TTA GCG GCA AAA AAA AAA AAA AAA AAA AAA AAA AAA AAA AGC CGC(T-sulfoCy3)/-3'

5'- / AAG CCT CGC TGC CGT CGC CA/-3'

Oligonucleotides for investigating the design rules

DNA structures were formed from the oligos below, as detailed in Supplementary Table 1.

3' labelling on T

1 5'- / GTGCCGCX/-3' (X = dT-sulfoCy3)

2 5'- / GTGCCGCX/-3' (X = T-phosphate-sulfoCy3)

3 5'- / GTGCCGCX/-3' (X = T-phosphate-Cy3)

4 5'- / AGCGGCAC/-3'

(complementary to **1-3** for forming end-labelled duplex)

5 5'- / GGCAGCGAGGCTTAGCGGCAC/-3'

(for overhang with **1-3** and nicked with **1-3** and **6**)

6 5' / AAGCCTCGCTGCC/-3'

(for nicked with **1-3** and **5**)

5' labelling on T

7 5'- / XCGCCGTG/-3' (X = dT-sulfoCy3)

9 5'- / XCGCCGTG (X = T-phosphate-Cy3)

10 5'- / CACGGCGA/-3'

(complementary to **7** and **9** for forming end-labelled duplex)

11 5'- / CACGGCGATTCGGAGCGACGG/-3'

(for overhang with **7-9** and nicked with **7-9** and **12**)

12 5'- / CCGTCGCTCCGAA/-3'

(for nicked with **7-9** and **11**)

3' labelling on A

13 5'- / GTGCCGCX/-3' (X = A-phosphate-sulfoCy3)

14 5'- / TGCGGCAC/-3'

(complementary to **13** for forming end-labelled duplex)

15 5'- / GGCAGCGAGGCTTTGCGGCAC/-3'

(for overhang with **13** and nicked with **13** and **6**)

3' labelling on C (used with **6** for nicked structure)

16 5'- / GTGCCGCX/-3' (X = C-phosphate-sulfoCy3)

17 5'- / GGCGGCAC/-3'

(complementary to **16** for forming end-labelled duplex)

18 5'- / GGCAGCGAGGCTTGGCGGCAC/-3'

(for overhang with **16** and nicked with **16** and **6**)

3' labelling on G (used with **6** for nicked structure)

19 5'- / GTGCCGCX/-3' (X = G-phosphate-sulfoCy3)

20 5'- / CGCGGCAC/-3'

(complementary to **19** for forming end-labelled duplex)

21 5'- / GGCAGCGAGGCTTCGCGGCAC/-3'

(for overhang with **19** and nicked with **19** and **6**)

Supplementary Tables

Table S1: Representative time-resolved fluorescence decay parameters donor-only hairpin H1 with sCy3 attached to T via the base (dT) at different NaCl concentrations: lifetimes (τ), fractional amplitudes (A), and the average amplitude-weighted lifetime (τ_{av}) are shown. The buffer was 20 mM Tris at pH 7.8.

Concentration	τ_1 (ns)	A_1 (%)	τ_2 (ns)	A_2 (%)	τ_3 (ns)	A_3 (%)	τ_{av} (ns)
0mM	1.51	10.6	0.64	34.8	0.22	54.6	0.50
20mM	1.85	20.7	0.80	34.4	0.24	45.0	0.76
50mM	1.92	31.1	0.90	36.3	0.24	32.6	1.00
100mM	2.00	32.4	0.96	39.5	0.24	28.1	1.09
200mM	2.05	32.3	1.07	40.2	0.28	27.5	1.17
500mM	2.11	34.6	1.10	41.5	0.29	23.9	1.26

Table S2: Representative time-resolved fluorescence decay parameters of hairpin HEG-3' with sCy3 attached to T via a phosphate at different NaCl concentrations: lifetimes (τ), fractional amplitudes (A), and the average amplitude-weighted lifetime (τ_{av}) are shown. The buffer was 20 mM Tris at pH 7.8.

Concentration	τ_1 (ns)	A_1 (%)	τ_2 (ns)	A_2 (%)	τ_3 (ns)	A_3 (%)	τ_{av} (ns)
0mM	2.47	28.7	1.37	35.2	0.33	36.1	1.31
20mM	2.25	44.0	1.04	31.0	0.24	25.0	1.37
50mM	2.38	37.2	1.29	38.2	0.31	24.6	1.46
100mM	2.24	47.3	1.01	31.3	0.25	21.4	1.43
200mM	2.23	47.7	1.01	33.9	0.24	18.4	1.45
500mM	2.26	44.6	1.17	36.9	0.31	18.5	1.50

Table S3: Representative time-resolved fluorescence decay parameters of hairpin HEG-5' dT with sCy3 attached to T via the base (dT) at different NaCl concentrations: lifetimes (τ), fractional amplitudes (A), and the average amplitude-weighted lifetime (τ_{av}) are shown. The buffer was 20 mM Tris at pH 7.8.

Concentration	τ_1 (ns)	A_1 (%)	τ_2 (ns)	A_2 (%)	τ_3 (ns)	A_3 (%)	τ_{av} (ns)
0mM	1.94	9.5	0.77	23.8	0.23	66.7	0.52
20mM	1.98	8.6	0.78	22.7	0.22	68.7	0.50
50mM	2.0	8.4	0.81	22.2	0.22	69.5	0.50
100mM	2.05	8.2	0.79	22.1	0.22	69.7	0.50
200mM	1.96	9.7	0.70	24.0	0.22	66.3	0.50
500mM	2.00	10.5	0.69	26.9	0.22	62.6	0.53

Table S4: Representative time-resolved fluorescence decay parameters duplex DNA with sCy3 attached to T via the base (dT) at different NaCl concentrations: lifetimes (τ), fractional amplitudes (A), and the average amplitude-weighted lifetime (τ_{av}) are shown. The buffer was 20 mM Tris at pH 7.8.

Concentration	τ_1 (ns)	A_1 (%)	τ_2 (ns)	A_2 (%)	τ_3 (ns)	A_3 (%)	τ_{av} (ns)
0mM	1.71	1.7	0.68	12.1	0.23	86.2	0.31
20mM	1.79	1.8	0.68	12.9	0.22	85.3	0.31
50mM	1.58	2.1	0.65	12.4	0.22	85.5	0.30
100mM	1.47	2.4	0.60	14.3	0.21	83.3	0.30
200mM	1.72	1.6	0.67	14.5	0.22	83.9	0.31
500mM	1.49	2.8	0.63	16.9	0.23	80.3	0.33

Table S5: DNA oligos and dye used for ssDNA samples and to form duplex, overhang and nicked structures. See SI text for details of each oligo sequence corresponding to oligos 1-21 in the table.

ssDNA	Dye	Duplex	Overhang	Nick
1	3' dT-sulfoCy3	1/4	1/5	1/5/6
2	3' T-phosphate-sulfoCy3	2/4	2/5	2/5/6
3	3' T-phosphate-Cy3	3/4	3/5	3/5/6
7	5' dT-sulfoCy3	7/10	7/11	7/11/12
9	5' T-phosphate-Cy3	9/10	9/11	9/11/12
13	3' dA-phosphate-sulfoCy3	13/14	13/15	13/15/6
16	3' dC-phosphate-sulfoCy3	16/17	16/18	16/18/6
19	3' dG-phosphate-sulfoCy3	19/20	19/21	19/21/6

Table S6. Representative time-resolved fluorescence decay parameters of ssDNA, dsDNA, overhang and nicked samples with sCy3 3′ dT upon the titration of NaCl: lifetimes (τ), fractional amplitudes (A), and the average amplitude-weighted lifetime (τ_{av}) are shown. See SI text for details of oligo sequence. The buffer was 20 mM Tris at pH 7.6.

**DNA sample (oligos)**	**NaCl (mM)**	**τ_1 (ns)**	**A1 (%)**	**τ_2 (ns)**	**A2 (%)**	**τ_3 (ns)**	**A3 (%)**	**τ_{av} (ns)**
ssDNA (1)	20	1.79	17.3	0.77	34.5	0.24	48.3	0.69
	50	1.73	20.2	0.73	35.0	0.22	44.8	0.70
	200	1.77	19.9	0.75	36.4	0.22	43.2	0.72
dsDNA (1+4)	20	1.78	28.0	0.92	36.4	0.25	35.7	0.92
	50	1.70	37.4	0.76	32.8	0.22	29.8	0.95
	200	1.85	38.5	0.83	34.1	0.22	26.6	0.99
overhang (1+5)	20	1.83	16.6	0.82	34.1	0.24	49.3	0.70
	50	1.79	17.2	0.76	36.0	0.22	46.8	0.68
	200	1.98	21.3	0.87	35.3	0.25	43.5	0.84
nicked (1+5+6)	20	2.20	18.1	0.97	31.6	0.26	50.3	0.84
	50	2.21	19.5	0.94	34.0	0.24	46.5	0.86
	200	2.37	28.5	1.07	30.2	0.25	41.4	1.10

Table S7. Representative time-resolved fluorescence decay parameters of ssDNA, dsDNA, overhang and nicked samples with sCy3 3′ phosphate upon the titration of NaCl: lifetimes (τ), fractional amplitudes (A), and the average amplitude-weighted lifetime (τ_{av}) are shown. See SI text for details of oligo sequence. The buffer was 20 mM Tris at pH 7.6.

**DNA sample (oligos)**	**NaCl (mM)**	**τ_1 (ns)**	**A1 (%)**	**τ_2 (ns)**	**A2 (%)**	**τ_3 (ns)**	**A3 (%)**	**τ_{av} (ns)**
ssDNA (2)	20	1.65	17.1	0.78	26.6	0.24	56.3	0.63
	50	1.69	19.2	0.80	29.1	0.24	51.7	0.68
	200	1.66	21.0	0.77	29.4	0.24	49.6	0.69
dsDNA (2+4)	20	1.59	36.3	0.85	31.2	0.23	32.4	0.92
	50	1.59	42.0	0.79	31.8	0.18	26.2	0.96
	200	1.56	43.0	0.80	32.8	0.21	24.3	0.97
overhang (2+5)	20	1.92	17.1	0.94	29.7	0.26	53.2	0.75
	50	1.92	23.8	0.85	34.8	0.21	41.4	0.84
	200	2.13	30.5	1.02	37.7	0.27	31.9	1.12
nicked (2+5+6)	20	2.45	39.7	1.27	22.1	0.29	38.2	1.36
	50	2.41	49.5	1.09	20.8	0.23	29.7	1.49
	200	2.47	48.8	1.27	18.9	0.26	32.3	1.53

Table S8. Representative time-resolved fluorescence decay parameters of ssDNA, dsDNA,overhang and nicked samples with Cy3 3′ phosphate upon the titration of NaCl: lifetimes (τ), fractional amplitudes (A), and the average amplitude-weighted lifetime (τ_{av}) are shown. See SI text for details of oligo sequence. The buffer was 20 mM Tris at pH 7.6.

**DNA sample (oligos)**	**NaCl (mM)**	**τ_1 (ns)**	**A1 (%)**	**τ_2 (ns)**	**A2 (%)**	**τ_3 (ns)**	**A3 (%)**	**τ_{av} (ns)**
ssDNA (3)	20	1.97	15.3	0.94	36.0	0.30	48.6	0.79
	50	1.97	13.4	0.94	36.3	0.31	50.2	0.76
	200	2.05	12.5	1.02	34.4	0.33	53.1	0.78
dsDNA (3+4)	20	1.68	17.4	0.78	33.8	0.30	48.8	0.71
	50	1.70	17.4	0.76	39.2	0.28	43.4	0.71
	200	1.61	18.0	0.66	45.5	0.22	36.5	0.67
overhang (3+5)	20	2.19	24.3	0.95	47.0	0.21	28.6	1.04
	50	2.18	26.5	0.94	45.7	0.24	27.8	1.07
	200	2.29	31.5	1.00	45.0	0.26	23.5	1.23
nicked (3+5+6)	20	2.21	38.7	1.01	39.3	0.33	21.9	1.33
	50	2.33	31.1	1.29	35.0	0.45	33.9	1.33
	200	2.32	27.1	1.29	37.2	0.43	35.8	1.26

Table S9. Representative time-resolved fluorescence decay parameters of ssDNA, dsDNA,overhang and nicked samples with sCy3 5′ dT upon the titration of NaCl: lifetimes (τ), fractional amplitudes (A), and the average amplitude-weighted lifetime (τ_{av}) are shown. See SI text for details of oligo sequence. The buffer was 20 mM Tris at pH 7.6.

DNA sample (oligos)	NaCl (mM)	τ_1 (ns)	A1 (%)	τ_2 (ns)	A2 (%)	τ_3 (ns)	A3 (%)	τ_{av} (ns)
ssDNA (7)	20	1.74	21.4	0.76	36.6	0.24	42.0	0.75
	50	1.69	21.3	0.72	37.7	0.22	41.0	0.72
	200	1.74	21.8	0.77	36.7	0.23	41.5	0.76
dsDNA (7+10)	20	1.80	41.3	0.90	28.5	0.27	30.2	1.08
	50	1.79	38.7	0.91	30.9	0.24	30.4	1.05
	200	1.75	42.2	0.84	31.2	0.22	26.6	1.06
overhang (7+11)	20	1.79	18.4	0.83	35.8	0.23	45.8	0.73
	50	1.94	19.4	0.85	38.6	0.22	42.0	0.80
	200	1.86	20.1	0.83	38.0	0.24	41.9	0.79
nicked (7+11+12)	20	1.91	8.9	0.81	23.7	0.23	67.4	0.51
	50	1.91	8.1	0.71	22.3	0.21	69.6	0.46
	200	2.01	6.9	0.68	22.9	0.21	70.2	0.44

Table S10. Representative time-resolved fluorescence decay parameters of ssDNA, dsDNA,overhang and nicked samples with Cy3 5′ phosphate upon the titration of NaCl: lifetimes (τ), fractional amplitudes (A), and the average amplitude-weighted lifetime (τ_{av}) are shown. See SI text for details of oligo sequence. The buffer was 20 mM Tris at pH 7.6.

DNA sample (oligos)	NaCl (mM)	τ_1 (ns)	A1 (%)	τ_2 (ns)	A2 (%)	τ_3 (ns)	A3 (%)	τ_{av} (ns)
ssDNA (9)	20	2.22	11.9	1.19	37.5	0.37	50.6	0.89
	50	2.21	11.0	1.19	35.8	0.37	53.2	0.87
	200	2.21	10.7	1.16	34.1	0.36	55.1	0.83
dsDNA (9+10)	20	1.67	4.5	0.68	42.6	0.31	52.9	0.53
	50	1.59	4.5	0.64	47.4	0.29	48.1	0.52
	200	1.50	4.5	0.66	40.9	0.31	54.5	0.51
overhang (9+11)	20	2.05	32.2	1.13	37.0	0.39	30.8	1.20
	50	2.05	33.8	1.09	38.0	0.37	28.2	1.21
	200	2.12	35.3	1.14	37.2	0.36	27.5	1.27
nicked (9+11+12)	20	2.07	26.3	1.05	40.7	0.42	33.0	1.11
	50	2.12	22.2	1.16	38.6	0.45	39.2	1.10
	200	2.13	21.4	1.10	41.5	0.44	37.0	1.08

Table S11. Representative time-resolved fluorescence decay parameters of ssDNA, dsDNA, overhang and nicked samples with sCy3 3′ phosphate attached to dA, dC and dG: lifetimes (τ), fractional amplitudes (A), and the average amplitude-weighted lifetime (τ_{av}) are shown. See SI text for details of oligo sequence. The buffer was 20 mM Tris at pH 7.6 and contained 20 mM NaCl.

DNA sample (oligos)	Base	τ_1 (ns)	A1 (%)	τ_2 (ns)	A2 (%)	τ_3 (ns)	A3 (%)	τ_{av} (ns)
ssDNA (13)	A	2.43	2.3	1.23	28.1	0.29	69.6	0.61
ssDNA (16)	C	1.49	20.6	0.64	31.2	0.20	48.1	0.61
ssDNA (19)	G	1.69	18.5	0.73	34.7	0.22	46.8	0.67
dsDNA (13+14)	A	1.78	16.6	0.84	40.5	0.25	42.9	0.69
dsDNA (16+17)	C	1.25	25.0	0.40	37.9	0.39	37.0	0.61
dsDNA (19+20)	G	1.15	19.2	0.62	49.0	0.24	31.8	0.60
overhang (13+15)	A	1.58	17.3	0.69	28.6	0.21	54.1	0.59
overhang (16+18)	C	1.66	19.2	0.74	33.7	0.21	47.1	0.67
overhang (19+21)	G	1.66	17.8	0.71	36.4	0.21	45.8	0.65
nicked (13+15+6)	A	2.46	27.0	1.20	18.3	0.26	54.7	1.02
nicked (16+18+6)	C	2.70	13.9	1.61	38.2	0.37	47.8	1.17
nicked (19+21+6)	G	2.40	47.2	1.12	16.8	0.24	35.0	1.41

Supplementary Figures

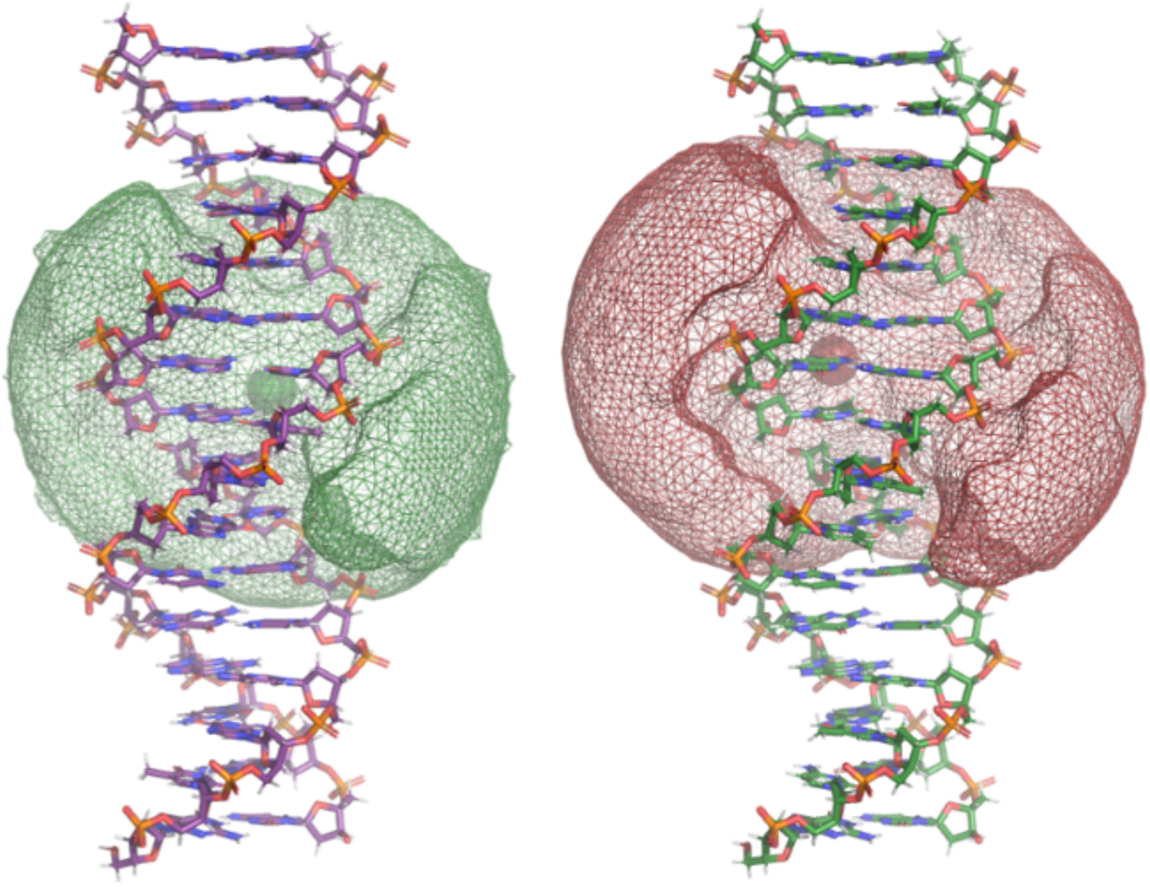

Figure S1. Model A (left) and model B (right) were created using x3dna and pymol. Model A has sCy3 attached via the dT linker at the 3′ end of the nick. The AV is shown as a green cloud around the duplex. Model B has the same dye attached at the 5′ end of the nick. The AV is shown as a red cloud around the duplex.
